# Testicular Gap (*CX43*) and Tight Junction (*OCLN*, *CLDN3*, *5* and *11*) Components in the Dog Are Affected by GnRH-Mediated Downregulation

**DOI:** 10.3390/ani16020254

**Published:** 2026-01-14

**Authors:** Sandra Goericke-Pesch, Lena Röhrs, Sven Wallrabenstein, Agnete Frimødt Rønnow, Daniela Fietz, Ralph Brehm, Marion Langeheine, Axel Wehrend, Bernd Hoffmann, Hanna Körber, Eva-Maria Packeiser

**Affiliations:** 1 Clinic for Obstetrics, Gynecology and Andrology of Large and Small Animals, Justus-Liebig-University Giessen, Frankfurter Str. 106, 35392 Giessen, Germanyaxel.wehrend@vetmed.uni-giessen.de (A.W.);; 2 Unit for Reproductive Medicine—Clinic for Small Animals, University of Veterinary Medicine Hannover, Foundation, Bünteweg 15, 30559 Hannover, Germany; hanna.koerber@tiho-hannover.de (H.K.); eva-maria.packeiser@tiho-hannover.de (E.-M.P.); 3 Department of Veterinary Sciences, Section for Veterinary Reproduction and Obstetrics, Faculty of Health and Medical Sciences, University of Copenhagen, Højbakkegård 5, 2630 Tåstrup, Denmark; 4 Institute of Veterinary Anatomy, Histology and Embryology, Justus-Liebig-University Giessen, Frankfurter Str. 94, 35392 Giessen, Germany; daniela.fietz@uni-giessen.de; 5 Institute of Anatomy, University of Veterinary Medicine Hannover, Bischofsholer Damm 15, 30173 Hannover, Germany; ralph.brehm@tiho-hannover.de (R.B.);

**Keywords:** blood–testis barrier, dog, GnRH-agonist, occludin, claudin, connexin 43

## Abstract

Following the downregulation of testicular endocrine and germinative function by slow-release gonadotropin-releasing hormone (GnRH)-agonist implants, testicular functions are quickly restored after implant removal. As an intact blood–testis barrier (BTB) is crucial for normal spermatogenesis and its integrity is FSH- and androgen-dependent, alterations in the BTB gene and protein expressions during downregulation and subsequent restart seem inevitable. We investigated *occludin* (*OCLN*), *claudin* (*CLDN*) *3*, *5*, *11,* and *connexin* (*CX*) *43* mRNA-, and CLDN11 and CX43 protein expressions during GnRH implant-induced downregulation (W0) and restart of spermatogenesis after implant removal (week, W, 3–12). Untreated juvenile (JG) and adult dogs (CG) served as controls. Sertoli cells were significantly affected by treatment (reduced nuclear area, *OCLN*, and *CLDN5* expressions). All investigated genes (except *CLDN3*) differed significantly during restart (W0–12) compared with CG (*p* < 0.05). CLDN11 and CX43 immunopositive staining was absent or diffuse cytoplasmic at downregulation and relocated at W9, indicating disruption and subsequent restorage of BTB. As W0 and JG differed considerably, our results suggest that the model cannot mimic puberty. In conclusion, GnRH implant-induced long-term gonadotropin suppression disrupts testicular CX43 and CLDN11 distribution and changes gap and tight junction mRNA expression. Treatment effects are reversible suggesting re-establishment of the BTB.

## Simple Summary

Slow-release gonadotropin-releasing hormone (GnRH)-agonist implants are used as a medical alternative to surgical castration, reversibly inducing basal testosterone and infertility in male dogs, with full recovery of testicular functions subsequent to implant removal. We hypothesized that the blood–testis barrier, essential for normal spermatogenesis, is reversibly affected by treatment. Gap and tight junction component expressions were studied at mRNA and protein level during efficient treatment and different weeks of recovery following implant removal and compared to untreated adult dogs. In relation to treatment, the blood–testis barrier was disrupted but recovered following recovery of spermatogenesis.

## 1. Introduction

The blood–testis barrier (BTB) is a physiological barrier essential for initiation and maintenance of spermatogenesis [1,2,3,4,5,6]. Its key functions include: 1. Compartmentalization into a basal and an adluminal compartment, to segregate the different developmental stages of spermatogenesis [7,8,9]; 2. Creation of a specific microenvironment; and 3. Immune protection; however, it is controversially discussed. The BTB is formed by Sertoli and adjacent germ cells and consists of several different types of junctions, including gap junctions, tight junctions, adherens junctions, ectoplasmic specializations, and desmosomes.

The gap junctional protein connexin 43 (CX43) represents the predominant testicular connexin of most species [2,4], including the dog [3]. Its localization is not restricted to adjacent Sertoli cells, it also connects Sertoli cells with germ and peritubular cells. Thereby, CX43 is essential for the cessation of proliferation and normal maturation of Sertoli cells, and the initiation of mitosis and meiosis in germ cells [10,11,12]. Furthermore, CX43 seems to regulate tight and adherens junction remodeling [13,14,15].

Constituents of tight junctions are the transmembranous occludin (OCLN), proteins of the claudin family (CLDN) and proteins of the junctional adhesion molecule (JAM) family. OCLN is involved in the regulation of paracellular diffusion and transportation [16,17], but it has no impact on the initiation and first appearance of functional tight junctions in knock-out mouse models [18,19]. The observation that OCLN expression is seasonal and stage-specific in the Djungarian hamster [20,21], but not in the mouse and dog [22,23] and possibly absent in the guinea pig and man [24], points to a species-specific role in BTB formation. CLDN3 regulates the progression of meiosis by promoting germ cell migration across the BTB [25], but is not associated with BTB permeability changes [6,21,25]. Different to that, CLDN11 is crucial for tight junction function as *CLDN11^−/−^* mice exhibit multiple deficits including male sterility due to severe changes in Sertoli cell structure and function [26]. Although the relevance of CLDN5 for the BTB is still unknown, its increased expression during the time of BTB formation gives hints to its involvement [27,28]. Investigations on claudins in canine testis are limited to *CLDN11* mRNA and CLDN3 and CLDN11 proteins in selected cases [29,30].

Gonadotropins, especially FSH (Follicle stimulating hormone), are physiological regulators of spermatogenic activity in rodents and other seasonal breeders [21,31,32,33,34]. However, the direction of expression alterations of BTB components are in part controversial: FSH, e.g., upregulates *CLDN11* mRNA expression and CLDN11 protein levels in the rat [31], but downregulates *CLDN11* mRNA expression in murine Sertoli cells [32]. It has also been shown that the expression of CLDN3 and CLDN11 is directly and positively influenced by testosterone in mice and rats [31,33,35,36]. In the dog, treatment with a slow-release gonadotropin-releasing hormone (GnRH)-agonist implant inhibits pituitary gonadotropin secretion, resulting in reliable suppression of the endocrine and germinative testicular function, with the whole steroidogenic cascade being affected and spermatogenesis being arrested at the level of spermatogonia/spermatocytes [37].

The aim of this study was to gain further insights into the hormonal regulation of gap and tight junction formation in the BTB. Therefore, gene expression of gap (*CX43*) and tight junction components (*CLDN3*, *5*, *11* and *OCLN*) as the most relevant BTB constituents in the literature were monitored in the downregulated canine testis and the process of recovery. The results were verified at protein level for CX43 and CLDN11. Here, a disrupted BTB, followed by a complete recovery was presumed. Further, the effects of three different GnRH implants were compared with each other, as well as with juvenile and untreated canine testes, expecting equal efficacy but clear differences in prepubertal conditions.

## 2. Materials and Methods

The study was conducted using tissue samples from a previous study [37,38]. Animal experimentation was approved by the respective authority (permit No. AZ V54-19c20/15c GI18/14, Regierungspräsidium Gießen). All dogs were owned by the university at the time of the study. Animals were, however, housed privately during the study period and presented at the university regularly at all study time points (for details see [37]). People taking the dogs into custody agreed by a written consent to follow the study design and to provide animals with a permanent home after castration at the end of the study. Collaboration with caretakers was excellent and all dogs were presented at all time points as agreed, indicating that this could be a suitable option for optimized life quality of research animals in specific non-final studies. All animals had been healthy during the study period, were regularly vaccinated and dewormed, suffered from no concomitant disease, and did not receive any other concomitant medication.

### 2.1. Experimental Design

The study included 27 sexually mature male Beagle dogs, that were submitted to a clinical and andrological examination. Parameters of semen analysis were within the normal range; thus, all dogs fulfilled the inclusion criteria [37,38].

**Figure 1:**
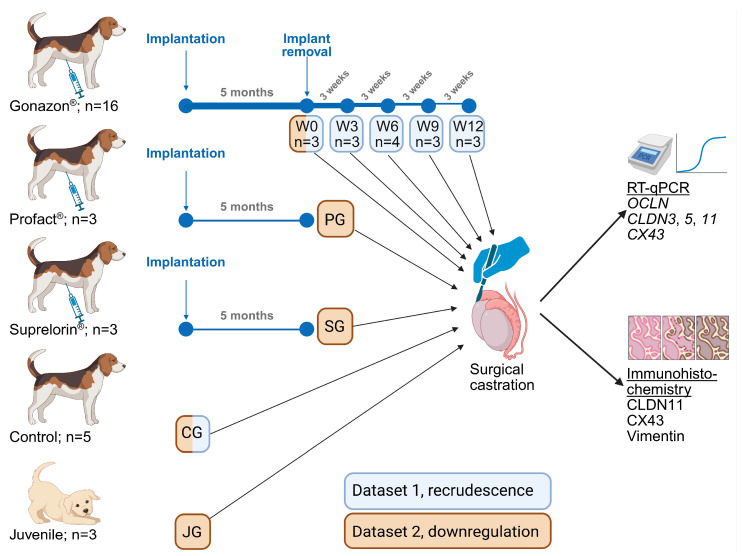
Study design. Dataset 1, recrudescence, was used to study recovery from 5 months treatment with an azagly-nafarelin GnRH implant (Gonazon^®^), and Dataset 2, downregulation, compares between the three different GnRH implants containing azagly-nafarelin (Gonazon^®^), buserelin (Profact^®^), and deslorelin (Suprelorin^®^), as well as with untreated adult and juvenile controls. Created in https://BioRender.com.

In Dataset 1, recrudescence, the recovery from GnRH slow-release implant treatment in Dataset 1 should be monitored. Therefore, 16 of these dogs received a treatment with a GnRH-agonist implant containing 18.5 mg azagly-nafarelin (Table 1, Figure 1, Gonazon^®^, Intervet, Angers, France) at the paraumbilical area. Five months later, the implants were removed under local anesthesia with basal testosterone concentrations [37], and the dogs were surgically castrated at the same day (W0, *n* = 3), or in 3-week intervals (week, W3 *n* = 3; W6 *n* = 4; W9 *n* = 3; W12 *n* = 3). Five of the remaining 11 Beagle dogs served as untreated control and were castrated directly at the beginning of the study.

In order to compare different GnRH implants in Dataset 2, downregulation (Figure 1, Table 1), the remaining six Beagle dogs received either a 6.3 mg buserelin acetate implant (PG, *n* = 3, Profact^®^ Depot; Sanofi-Aventis, Frankfurt Hoechst, Germany), or a 4.7 mg deslorelin implant (SG, *n* = 3, Suprelorin^®^; Virbac, Bad Oldesloe, Germany) [38]. All dogs were castrated at full downregulation five months after implantation with basal serum testosterone levels. Additionally, testes from three untreated juvenile mixed breed males aged 2.5 months (group JG) [37,38] were obtained (Table 1, Figure 1, Dataset 2). Dogs in JG had been selected by health status and age, but no other criteria. For comparative reasons, W0 and CG from Dataset 1 were also included in Dataset 2.

Testes tissue samples of approximately 0.5 cm^3^ size were collected and either stored at −80 °C until RNA extraction or fixed in Bouin’s solution and embedded in paraffin blocks for immunohistochemistry as previously described in detail [37].

### 2.2. RNA Isolation and Quantitative Real Time PCR (RT-qPCR)

Total RNA from RNAlater^®^-immersed testis samples was isolated using TRIzol^®^-Reagent (Life Technologies, Darmstadt, Germany) according to the manufacturer’s instructions. The RNA concentration was assessed photometrically (Eppendorf AG, Hamburg, Germany). Reverse transcription was performed using 200 ng DNase-treated RNA/µL and the GeneAmp Gold RNA PCR Kit (Perkin-Elmer Applied Biosystem GmbH, Weiterstadt, Germany) in accordance with the manufacturer’s protocols. Primers (Table 2) were designed using the freeware Oligo Explorer 1.5 (Gene Link Inc., Elmsford, NY, USA) and purchased from biomers.net GmbH (Ulm, Germany). *Glyceraldehyde-3-phosphate dehydrogenase* (*GAPDH*) was chosen as reference gene as it was superior to *β-actin* in previous experiments on the same sample set [39,40]. The specificity of all primers was checked using BLAST version 2.9.0 [41] (http://blast.ncbi.nlm.nih.gov, accessed on 15 November 2019) and results were confirmed by sequencing of PCR products (SRD GmbH, Bad Homburg, Germany).

RT-qPCR was performed in 20 µL reactions using 1 µL cDNA and iQTM SYBR Green Supermix (BioRad Laboratories GmbH, München, Germany). RT-qPCR conditions were 95 °C for 3 min, followed by 43 cycles of 95 °C for 10 s, 60 °C for 1 min, and 95 °C 10 s. All samples were run in duplicates using a CFX96TM real-time PCR system (BioRad Laboratories GmbH, München, Germany) and a no-template control was included in every assay. PCR efficiencies (Table 2) were calculated by using a relative standard curve derived from triplets of a 10-fold dilution series of pooled cDNA samples [42]. Evaluation of the gene expression ratios was an efficiency-corrected relative quantification according to Pfaffl [42].

### 2.3. Immunohistochemistry and Evaluation of Connexin 43, Claudin 11, and Vimentin

On (3–4 µm) sections from Bouin-fixed blocks, an immunoperoxidase method was applied. Briefly, after deparaffinization, antigen retrieval by boiling in citrate buffer (pH 6.0), quenching of endogenous peroxidase, several washing steps with Tris-HCl, and blocking of unspecific binding with 10% goat serum, the slides were overlaid with primary antibodies against CX43 (#3512, Cell Signaling Technology^®^ Inc., Frankfurt/Main, Germany, 1:50) or Claudin11 (ab53041, Abcam, Cambridge, UK, 1:2000) overnight at 4 °C. Specific primary antibody binding was confirmed previously [29,43]. Controls were incubated with the irrelevant rabbit IgG (I-1000, Vector^®^ Laboratories, Newark, CA, USA) instead of the respective primary antibody at an equal concentration. For CX43, the protocol was continued with secondary biotinylated goat anti-rabbit antibody (VECTASTAIN^®^ PK-6101 Rabbit IgG Elite ABC Kit, Vector Laboratories), followed by the Vector^®^ Nova-RED™ substrate kit (Vector Laboratories), all according to the manufacturer’s instructions. The EnVisionTM + Kit HRP Rabbit DAB + (Dako, Hamburg, Germany, K4011) was used for the Claudin 11 immunostaining. For descriptive analysis, representative slides were counterstained with Mayer’s hematoxylin (Carl Roth, Karslruhe, Germany), while counterstaining was omitted in those slides used for computer-assisted evaluation.

Slides were visually assessed at 400-fold magnification for presence, intensity, and localization of staining in at least 20 approximately round tubules per dog. Images were taken at 400-fold magnification on a Leica IM microscope (Leica, Wetzlar, Germany) by one observer, and the subsequent evaluation of intratubular signals was performed via a computer-assisted image analysis using ImageTool 3.0, freeware (UTHSCSA, San Antonio, TX, USA, University of Texas, https://imagetool.software.informer.com/3.0/, accessed on 15 November 2019). The interstitium was excluded from evaluation by setting the region of interest to the tubules only. The spermatogenic stages within the tubules were selected to represent the composition within the respective sample, and tubules with unspecific color reactions were excluded. Images were transformed into a gray scale version, pixels with the gray scale 0 were defined as white and 255 as black, and individual thresholds for immunopositive staining were defined for CX43 (115) and Claudin 11 (56). Mean gray scales of the pixels within the defined immunopositive area per tubule were calculated and the percentage of immunopositive area (PIA) was determined by transforming all immunostained pixels to black (binarization) and dividing the number of stained pixels by the total number of pixels inside the tubules.

For an evaluation of possible stage-specific immunostaining, the slides of all dogs that showed complete spermatogenesis were analyzed in a meandering pattern, and tubules that were approximately round in shape with a distinct lumen were evaluated. As described by Russell et al. 1990 [44], these tubules were classified into the individual stages (I–VIII) of spermatogenesis and assessed according to the location and intensity of the immunohistological signal present.

Vimentin staining was conducted similarly, with the following modifications: Unspecific binding sites were blocked with 10% horse serum and a primary monoclonal vimentin antibody clone V9 (#GA63061-2, Dako Deutschland GmbH, Hamburg, Germany; 1:2000) or an irrelevant mouse IgG control antibody (I-2000, Vector^®^ Laboratories) was applied overnight, followed by a horse anti-mouse secondary antibody (BA-2000, Vector^®^ Laboratories, 1:200). With the AEC substrate kit (BioPrime, BioLogo, Kronshagen, Germany), the staining was made visible. Nuclei were counterstained with Mayer’s hematoxylin (Carl Roth).

Specific cytoplasmic vimentin staining of Sertoli cells, adjacent to the basal membrane, allowed us to determine the area of 100 Sertoli cell nuclei per dog in ten visual fields of a 200× magnification using a Leica IM1000 software, version V.1.20 (Leica) with procedure as previously described for Leydig cell nuclei [37]. Ovoid nuclei were included with at least one nucleolus. Right-angled nucleic diameters were measured and the Sertoli cell nuclear area was calculated as ¼ × length × width × π. Means of the 100 measured nuclei were calculated.

### 2.4. Statistical Analysis

At first, the data was tested for normal distribution with the Shapiro–Wilk test. For nearly normally distributed data (mean gray scale), the arithmetic mean and standard deviation ($\bar{x}$ (SD)) are given. In case of an uneven distribution, logarithmical transformation of data was applied (ratio *CX43*, *OCLN*, *CLDN3*, *5*, *11*) and data are presented as geometric mean and geometric standard deviation ($\bar{x}$_g_ (SD_g_)). As PIA for CX43 and CLDN11 was distributed skewed to the right, arc sine transformation of data was necessary for statistical analysis to obtain an approximately normal distribution. Mean values and SD of these arc sine-transformed data were calculated and the data retransformed, yielding the modified mean ($\bar{x}$_r_) and the range of mean ± 1 SD [45,46].

Two groups of data are compared (Table 1, Figure 1): Data set 1 (recrudescence) consisted of the groups reflecting recovery of spermatogenesis compared to untreated adult controls (groups W0, W3, W6, W9, W12, and CG). Data set 2 (downregulated testis) consisted of the groups reflecting the state of downregulation with different GnRH-agonist implants compared to juvenile and adult untreated controls (W0, PG, SG, CG, and JG). To test for the effect of group, a one-way analysis of variance (ANOVA) was applied, followed by the Tukey–Kramer comparison test in case the results of the ANOVA indicated a significant effect of group.

For all tests, the statistical software package BMDP Release 8.1 (BMDP Statistical Software, Inc., Cork, Ireland) was used. Values were considered to be statistically significant at a level of *p* < 0.05.

## 3. Results

### 3.1. Changes in Tight Junction Components—Expression of Occludin

**Figure 2:**
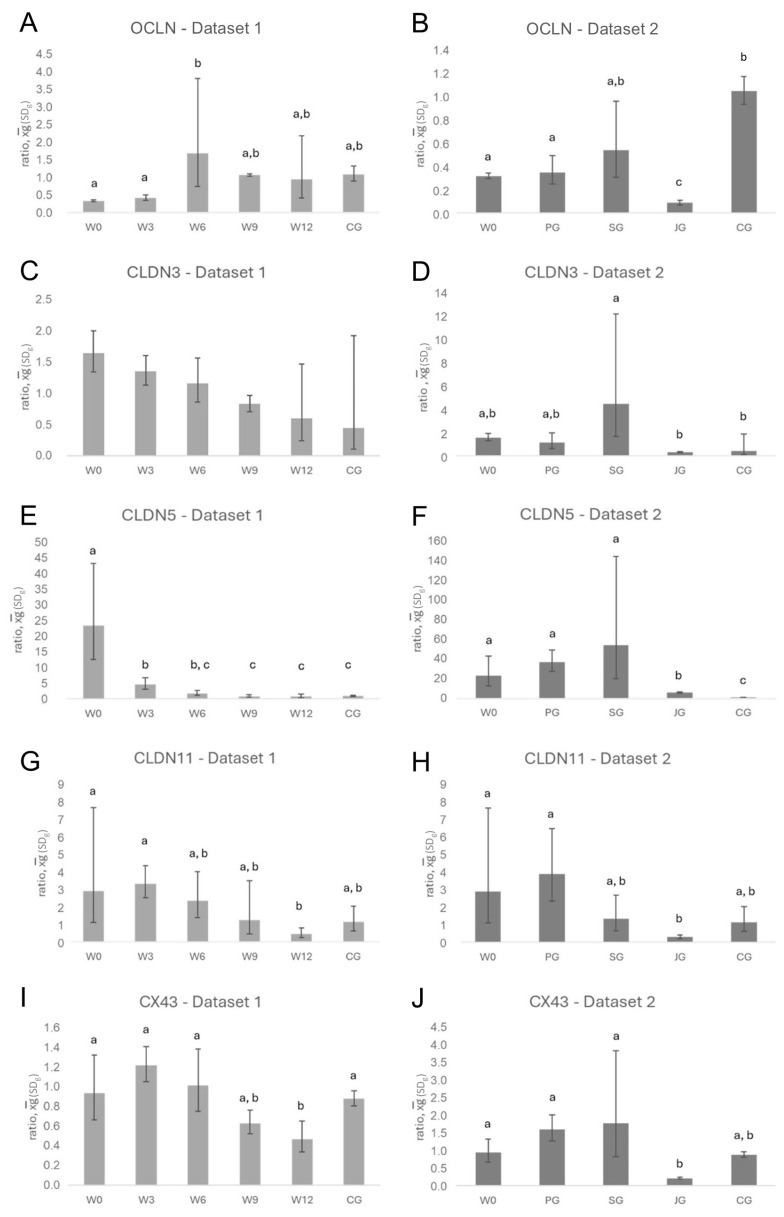
Relative gene expression (ratio) in canine testicular tissue during recrudescence of spermatogenesis (week 0 to 12) compared to adult controls (CG) (Dataset 1; (**A**,**C**,**E**,**G**,**I**)) and in the downregulated canine testis using different GnRH agonists (W0, PG, and SG) compared to juvenile (JG) and adult controls (CG) (Dataset 2; (**B**,**D**,**F**,**H**,**J**)). Presented are (**A**)/(**B**)**:** *occludin* (*OCLN*), (**C**)/(**D**)**:** *claudin* (*CLDN*) *3*, (**E**)/(**F**): *CLDN 5* and (**G**)/(**H**): *CLDN 11*, as well as (**I**)/(**J**): *Connexin 43* (*CX43*) mRNA Results are presented as $\bar{x}$_g_ (SD_g_); different letters indicate significant differences between groups.

Results of RT-qPCR for *OCLN* revealed a significant effect of group in the recrudescence Dataset 1 (ANOVA, *p* = 0.0074) with lowest ratios observed in W0 and W3. The W6 ratio was higher than in groups W0 and W3 (Tukey test, both *p* < 0.05), and also higher than in W9, W12, and CG, although without significance (Figure 2A). Similarly, the downregulated groups differed significantly (ANOVA, *p* = 0.0001, Figure 2B) with the ratio being lower in JG compared to all other groups (Tukey test, *p* < 0.01). The ratios of W0 and PG were lower than in CG (Tukey test, *p* < 0.01).

### 3.2. Changes in Tight Junction Components—Expression of Claudin 3, 5, and 11

During recrudescence, the expression of all three claudin genes decreased from W0 to a level comparable with CG in W12. The decrease was steady, but without significance for *CLDN3* (Figure 2C). In contrast, the ANOVA revealed a significant effect of group for *CLDN5* and *11* expressions (*p* < 0.0001 and *p* = 0.0204, respectively, Figure 2E,G). *CLDN5* expression was significantly higher in W0 compared to all other groups (Tukey test, *p* < 0.01) and still significantly higher in W3 compared to W9, W12, and CG (Tukey test, *p* < 0.01), but normalized fastest (Figure 2E). *CLDN11* expression was higher at downregulation (W0) and W3 compared to W12 (Tukey test, *p* < 0.05) with no further significant differences identified (Figure 2G). Comparing the three different GnRH agonists, ANOVA revealed a significant effect of group for the mRNA expression of *CLDN3* (*p* = 0.0292), *CLDN5* (*p* < 0.0001), and *CLDN11* (*p* = 0.0034) (Figure 2D,F,H), with all three claudin genes tending to be upregulated at suppressed spermatogenesis. *CLDN3* expression of SG was significantly higher than the one of CG and JG (Tukey test, *p* < 0.05). Regarding *CLDN5*, mRNA expression in W0, PG, and SG were significantly higher compared to CG (Tukey test, *p* < 0.01) and JG (Tukey test, *p* < 0.01–0.05), with the ratio in JG being higher than in CG (Tukey test, *p* < 0.01). *CLDN11* expression was highest in W0 and PG and lowest in JG (Tukey test, *p* < 0.01).

**Figure 3:**
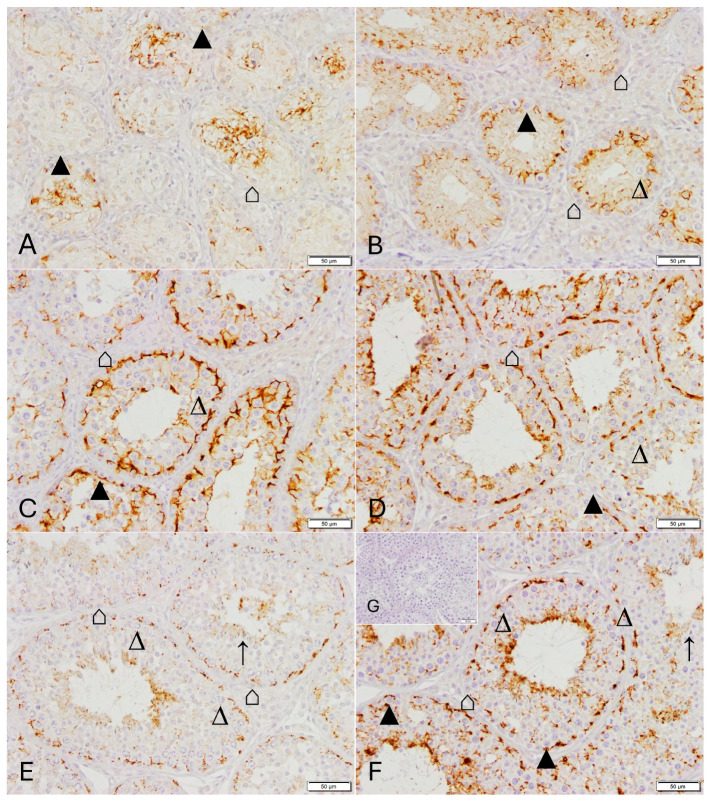
Immunostaining for Claudin 11 in dog testicular tissue Dataset 1. (**A**): group W0, (**B**): group W3, (**C**): group W6, (**D**): group W9, (**E**): group W12, (**F**): CG (control group), (**G**) (insert): negative control (all magnification: ×200, scale bar = 50 µm). ⌂ Sertoli cell, ▲ spermatogonia, ∆ primary spermatocyte, ↑ elongating spermatids.

**Figure 4:**
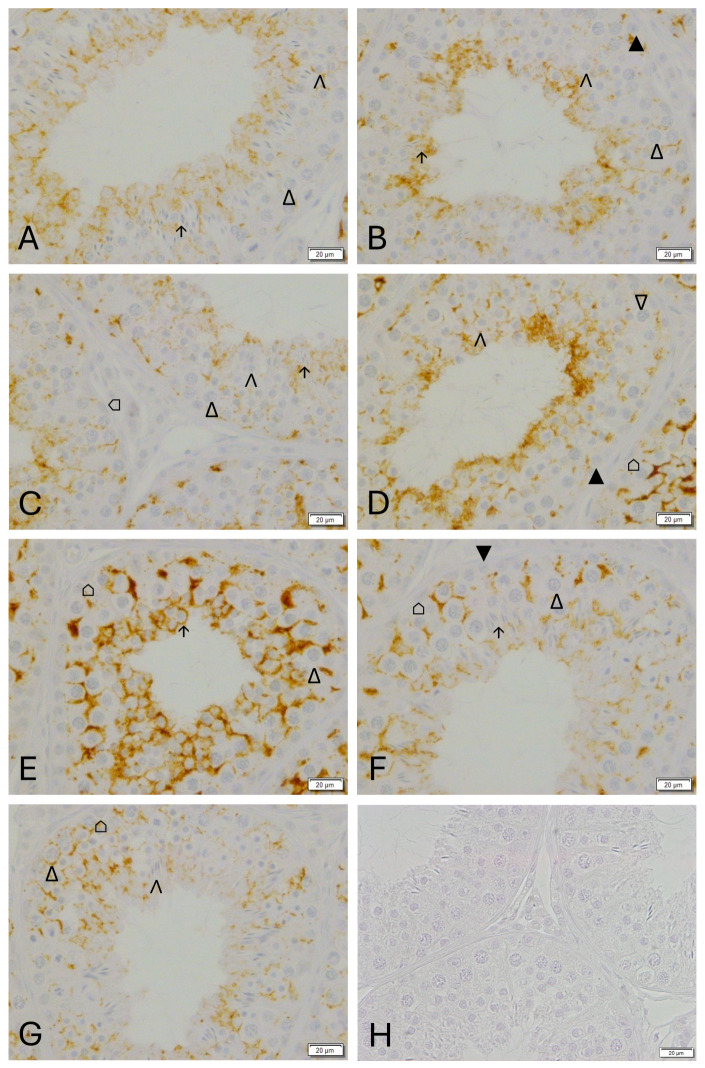
Spermatogenic stage-specific immunostaining for Claudin 11 in dog testicular tissue in the control group (CG): (**A**): stage I, (**B**): stage II-III, (**C**): stage IV, (**D**): stage V, (**E**): stage VI, (**F**): stage VII, (**G**): stage VIII, (**H**): negative control (all magnification: ×400, scale bar = 20 µm). ⌂ Sertoli cell, ▲ spermatogonia, ∆ primary spermatocyte, ∧ round spermatids, ↑ elongating spermatids.

**Figure 5:**
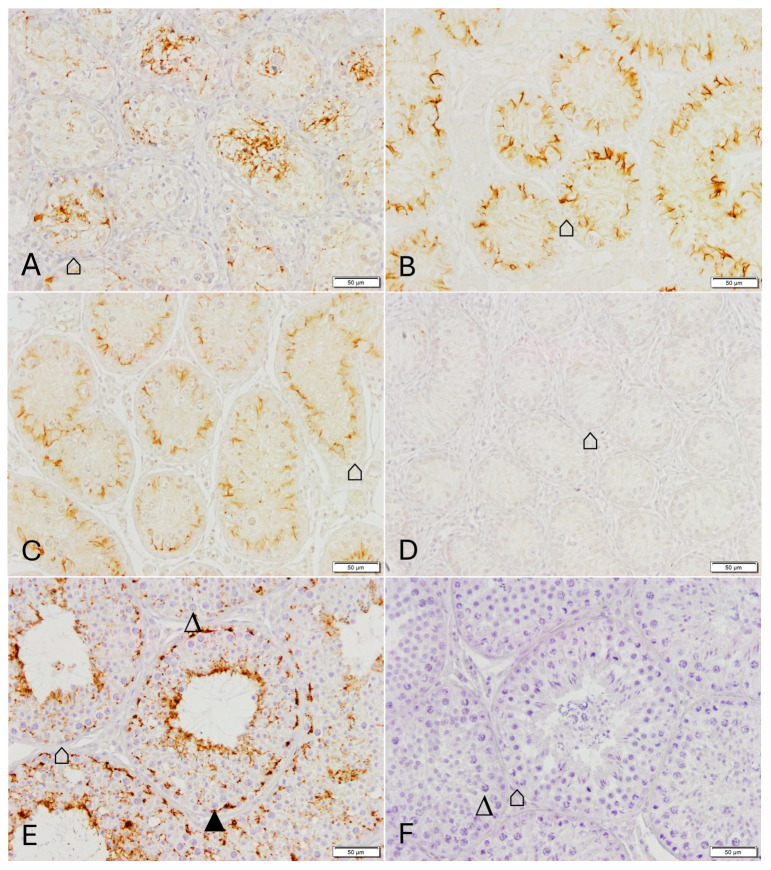
Immunostaining for Claudin 11 in dog testicular tissue Dataset 2. (**A**): group W0, (**B**): PG; (**C**): SG, (**D**): JG (juvenile), (**E**): CG (control group), (**F**): negative control (all magnification: ×200, scale bar = 50 µm). ⌂ Sertoli cell, ▲ spermatogonia, ∆ primary spermatocyte.

Positive CLDN11 immunostaining was restricted to the Sertoli cells (Figure 3, Figure 4 and Figure 5, Figures S1 and S2). It was variable in W0 with some tubules showing a distinct staining between Sertoli cells, and, between Sertoli cells and spermatogonia (Figure 3A and Figure S1A), some tubules showing no staining and others showing a diffuse staining in the supranuclear tubular area. Staining was located above the spermatogonia and adluminal in W3 (Figure 3B and Figure S1B). From W6 onwards, a specific staining was visualized in the basal compartment typically indicating the BTB (Figure 3C and Figure S1C). An obvious filamentous staining could be detected between adjacent Sertoli cells, and Sertoli and germ cells (spermatogonia and primary spermatocytes) from W9 onwards (Figure 3D,E and Figure S1D,E). In tubules with elongated spermatids, an additional adluminal staining “line” was observed (Figure 3D–F and Figure S1D–F). Additionally, an intermediary compartment with a distinct staining between basal and adluminal staining pattern could be identified. The same pattern with a distinct filamentous staining was observed in CG (Figure 3F and Figure S1F).

In normal spermatogenesis, CLDN-11 was expressed in Sertoli cell cytoplasm in all stages (Figure 3 and Figure 4). The expression varied depending on the cellular composition of stages, with two (basal versus adluminal) or three (basal—intermediate—adluminal) compartments being identified: A regular Sertoli cell staining occurred between spermatogonia and spermatocytes (stages I, V, Figure 4A,D), spermatocytes and round spermatids (stage II and III, IV, Figure 4B,C), spermatocytes and elongating spermatids (stage I, VI, Figure 4A,E), as well as between spermatocytes and spermatocytes (stage VI and VII, Figure 4E,F). There were no obvious differences in staining intensity. In addition, an adluminal staining in the Sertoli cell cytoplasm was detected, with it being subjectively strongest in stages V, VI, and VII (Figure 4E,F).

In the quantitative evaluation of CLDN11-positive staining, PIA did not differ significantly between groups. The mean gray scale value showed a tendency (ANOVA, *p* = 0.0792) with the lowest values in W0 (Table 3).

No immunopositive CLDN11 staining was detected in JG (Figure 5D and Figure S2D), whereas the staining was variable within the Sertoli cell cytoplasm at downregulation independent of the GnRH agonist used (Figure 5A–C and Figure S2A–C). There was a significant effect of group for PIA (ANOVA, *p* = 0.0179) and the mean gray scale values (ANOVA, *p* = 0.0008), both parameters were significantly lower in JG compared to CG (PIA) and compared to all other downregulated groups and CG (mean gray scale) (Table 3).

### 3.3. Changes in Gap Junction Components—Expression of Connexin 43

At the mRNA level, there was a significant effect of group within the recrudescence Dataset 1 (ANOVA, *p* = 0.0024, Figure 2I) with the highest *CX43* ratio observed in W3. Interestingly, the *CX43* expression in W12 was significantly lower compared to W0, W3, W6, and CG (Tukey test, *p* < 0.05). In dataset 2, the ANOVA yielded a significant effect of group for the *CX43* mRNA-ratio (*p* = 0.0001, Figure 2J); the ratio was lowest in JG and different from W0 (Tukey test, *p* < 0.05), PG and SG (Tukey test, *p* < 0.01) but not from CG.

**Figure 6:**
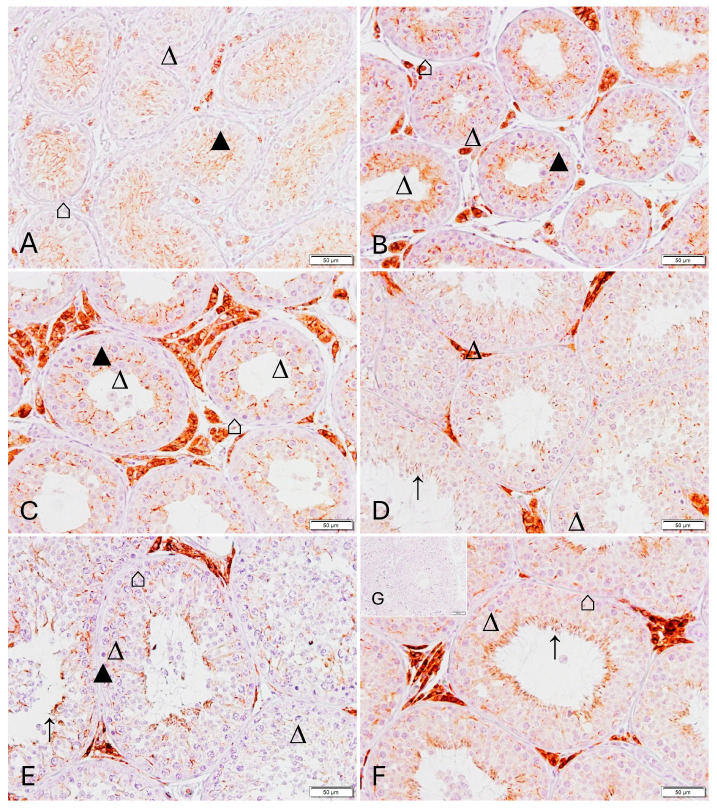
Immunostaining for Connexin 43 in dog testicular tissue Dataset 1. (**A**): group W0, (**B**): group W3, (**C**): group W6, (**D**): group W9, (**E**): group W12, (**F**): CG (control group), (**G**) (insert): negative control (all magnification: ×200, scale bar = 50 µm). ⌂ Sertoli cell, ▲ spermatogonia, ∆ primary spermatocyte, ↑ elongating/elongated spermatids.

A strong positive CX43 immunostaining was observed in the interstitial Leydig cells with no apparent difference between groups (Figure 6 and Figure S3). Concerning the Sertoli cells in W0, the immunopositive signal was diffuse and particularly located in the supranuclear area within the Sertoli cell cytoplasm (Figure 6A and Figure S3A). Due to the formation of a tubular lumen in W3, the staining was located in the adluminal region (Figure 6B and Figure S3B). From W6 onwards, a definite staining was also observed in the basal compartment of the seminiferous epithelium (Figure 6C and Figure S3C). An obvious staining could be detected between adjacent Sertoli cells, and between Sertoli and germ cells (spermatogonia and primary spermatocytes) from W9 onwards (Figure 6D and Figure S3D). In tubules with elongated spermatids, an additional adluminal staining “line” was observed (Figure 6E and Figure S3E). The same filamentous staining pattern was observed in CG (Figure 6F and Figure S3F).

**Figure 7:**
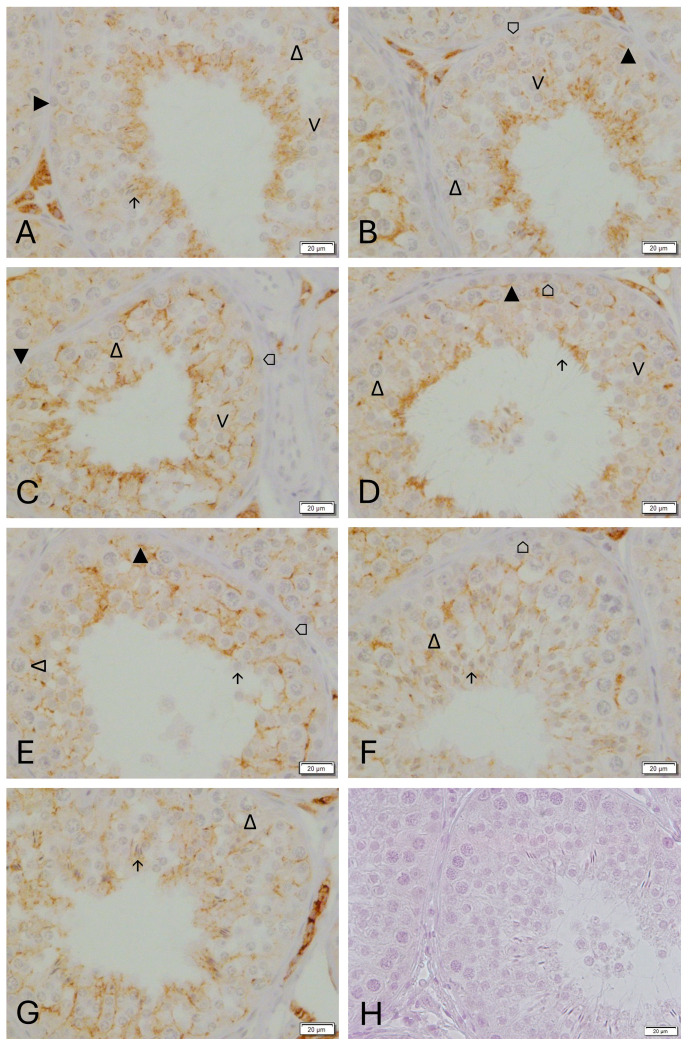
Spermatogenic stage-specific immunostaining for Connexin 43 in dog testicular tissue in the control group (CG): (**A**): stage I, (**B**): stage II-III, 3, (**C**): stage IV, (**D**): stage V, (**E**): stage VI, (**F**): stage VII, (**G**): stage VIII, (**H**): negative control (all magnification: ×400, scale bar = 20 µm). ⌂ Sertoli cell, ▲ spermatogonia, ∆ primary spermatocyte, ∧ round spermatids, ↑ elongating spermatids.

CX43 was regularly found in Sertoli cells with an indication of stage-specific expression in normal spermatogenesis (CG, Figure 6 and Figure 7). Staining was located in the basal compartment between Sertoli cells and spermatocytes and in the adluminal compartment between Sertoli cells and round and elongated spermatids. Overall, the staining intensity was highest in stage IV (Figure 7C) representing the stage before fully elongated spermatids were visible. Intensity was lowest in stage VI and VII (Figure 7E,F), representing the stages with two layers of primary spermatocytes. The adluminal signal was strongest in stage V (Figure 7D) before spermiation.

**Figure 8:**
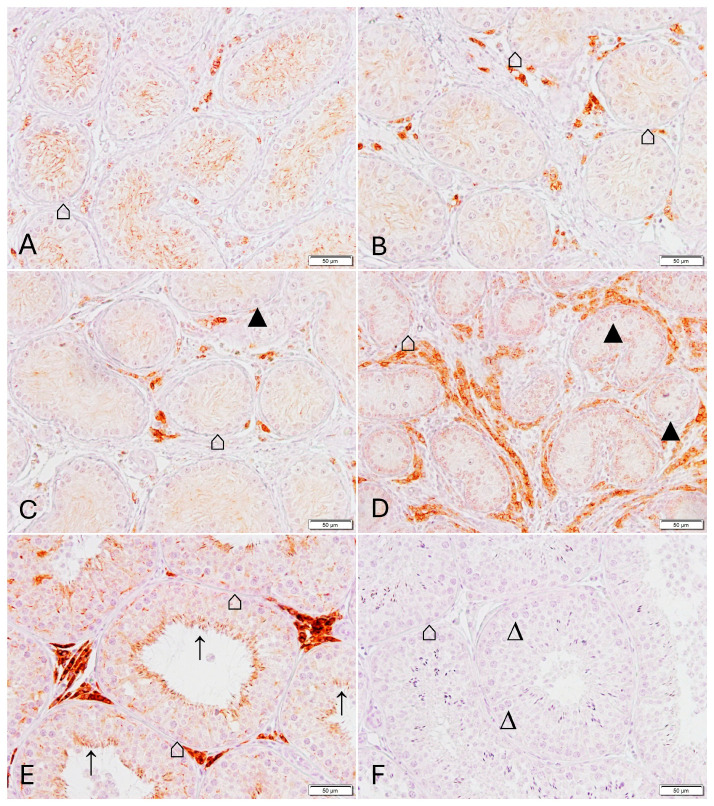
Immunostaining for Connexin 43 in dog testicular tissue Dataset 2. (**A**): group W0, (**B**): PG, (**C**): SG, (**D**): JG (juvenile), (**E**): CG (control group), (**F**): negative control (all magnification: ×200, scale bar = 50 µm). ⌂ Sertoli cell, ▲ spermatogonia, ∆ primary spermatocyte, ↑ elongating spermatids.

In immunohistochemistry, there was also a distinct CX43 staining of Leydig cells in all groups of Dataset 2 (Figure 8 and Figure S4). Diffuse immunopositive signals within the Sertoli cell cytoplasm were observed at downregulation independent of the GnRH agonist used (Figure 8A–C and Figure S4A–C). Although a similar staining pattern with the immunopositive signal restricted to the Sertoli cell cytoplasma was observed in JG, staining intensity was higher in the periphery of the tubules (Figure 8D and Figure S4D).

Concerning the intratubular staining quantification, there was a significant effect of group for PIA (ANOVA, *p* = 0.0021) and mean gray scale values (ANOVA, *p* = 0.0028) in Dataset 1, recrudescence. PIA was significantly (Tukey test, *p* < 0.01) higher in W3 compared to W12 and CG (Table 3). The mean gray scale value was significantly lower in W6 compared to W0, W3 and CG (Tukey test, *p* < 0.05), and in W12 compared to W0 (Tukey test, *p* < 0.05, Table 3). In dataset 2, there was an effect of group for PIA (ANOVA, *p* = 0.016); it was highest in JG and significantly different from PG and CG (Tukey test, *p* < 0.05), while there were no differences between CG and the treatment groups (Table 3). The mean gray scale values showed no differences.

### 3.4. Area of Sertoli Cell Nuclei

**Figure 9:**
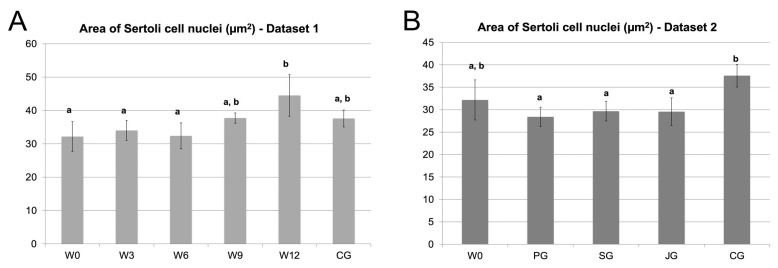
The area of Sertoli cell nuclei (in µm^2^) in (**A**). Dataset 1: recrudescence of spermatogenesis following treatment with a slow-release GnRH-agonist implant containing azagly-nafarelin and (**B**). Dataset 2: comparing downregulated, juvenile (JG), and adult testes (CG). Downregulation was achieved with three different GnRH-agonist implants. Data are presented as $\bar{x}$ (SD); different letters indicate significant differences between groups; W0–12: castration 0–12 weeks after Gonazon^®^ implant removal; CG: control group; PG: Profact^®^ group; SG: Suprelorin^®^ group; JG: juvenile group.

There was a significant effect of group for Dataset 1 (recrudescence) (ANOVA, *p*= 0.0072, Figure 9A) and 2 (downregulated testis) (ANOVA, *p* = 0.0047, Figure 9B). During recrudescence the area of Sertoli cell nuclei increased from W0 to W12; nuclear size was significantly smaller in W0, W3, and W6, compared with W12 (Tukey test, *p* < 0.05 each, Figure 9A). Concerning the downregulated testes (dataset 2, Figure 9B), there were no differences between groups W0, PG, SG, and JG, but the area of Sertoli cell nuclei was significantly higher in CG when compared to PG (Tukey test, *p* < 0.01), SG and JG (Tukey test, *p* < 0.05 each).

## 4. Discussion

### 4.1. Changes During Recrudescence of Spermatogenesis Compared to the Adult Controls, CG

The restart of spermatogenesis, the so-called testicular recrudescence, significantly affected all investigated BTB parameters, except for *CLDN3*. Whereas *OCLN* was low at downregulation and increased afterwards, with the highest level in week six, *CLDN5*, and, by trend, also *CLDN3* were highest at downregulation and decreased during the first six weeks, remaining constant thereafter. Similarly, *CLDN11* and *CX43* were higher initially and lowest in week twelve. However, when interpreting these data, the altered histological composition has to be considered [47], with an arrest of spermatogenesis at the level of spermatogonia/primary spermatocytes and a significantly reduced area of the tubular compartment at downregulation resulting in a relative enrichment of Sertoli and Leydig cells. BTB components, which are not expressed by spermatids and spermatocytes but by Sertoli and, in the case of CX43, also by Leydig cells, might thus be enriched in whole tissue lysates with arrested spermatogenesis, even without being actively regulated. The absence of significant differences in CLDN11 protein expression further supports a rather unaffected claudin expression. However, in view of this assumption, particularly the opposite effect observed at downregulation (W0) for the ratios for *OCLN* might be indicative of an active, inhibitory regulatory mechanism at downregulation. Investigations at mRNA-level following laser-assisted cell picking of the tubules or single cell sequencing could provide further clarification. Additionally, the discrepancy observed between CLDN11 mRNA and protein expression can be partly explained by decoupling of protein transcription and translation, as it is well known for other proteins in spermatogenesis, such as protamines and the androgen receptor [38,48].

At protein level, only synthesis and spatio-temporal immunolocalization of CX43 and CLDN11 were assessed due to the lack of specific antibodies for canine testicular tissues. Namely, three commercially available antibodies against OCLN were tested, but failed to obtain specific results in immunohistochemistry and Western blotting. Concomitant with basal/low LH, FSH, and testosterone concentrations [37], CX43 expression was significantly altered with a variably spread staining and an increased PIA, indicating disrupted or at least altered BTB function at downregulation and recrudescence. Interestingly, CX43 expression was still altered in week three, despite hormone levels being recovered indicating a delayed response and restoration [37]. Nevertheless, during re-establishment of spermatogenesis, a re-organization to a filamentous staining, especially at the basal compartment comparable to CG, confirmed recovery. Even if not significant, a similar observation was made for CLDN11 staining. These results support the well described regulatory effect of FSH on tight and gap junction protein expression in rats [31,49] and mice [50]. However, as FSH is less affected by GnRH-agonist treatment compared to LH, also in our canine testis model, these results might also further support the recent observations about the hormonal control of Sertoli cell activity, even in adulthood [51]. Interestingly, the heterogenous expression pattern of CLDN11 protein at downregulation—similar to altered human and canine spermatogenesis [30,52]—possibly reflects different states of BTB functionality in individual tubules, as postulated in hamsters and rats [53]. The observation that the tight junction protein CLDN11 does not seem to be as severely affected as CX43, indicated by BTB-specific immunostaining in some tubules, deserves further investigations.

Germ cells are embedded in a stage-specific manner in the Sertoli cell cytoplasm. Different to rodent spermatogenesis, canine spermatogenesis is less structured, similar to human spermatogenesis. Applying the staging according to Russell [44], we identified a stage-specific expression of CX43 in normal canine spermatogenesis, as previously described in other species [2,4,54]. The expression of the investigated BTB components at mRNA and protein level points to a disintegration of the BTB during downregulation. Reorganization of the BTB occurs rapidly with no significant difference being detected following recrudescence (W12), compared with the untreated adult controls. The stage-specific staining with a significantly lower immunopositive signal in stages VI and VII points to a partial disintegration of the BTB for migration of preleptotene spermatocytes from the basal to the adluminal compartment.

Establishment of a tubular lumen and basal position of the Sertoli cell nuclei are—among other signals—indicators of Sertoli cell polarity. Polarization of the Sertoli cell and establishment of occluding zonules contribute to the milieu needed for spermatogenesis [6,55,56] and are, consequently, important for an intact BTB [49] and, hence, spermatogenesis. Similarly to earlier observations indicating restoration of spermatogenesis nine weeks after implant removal [37], the area of Sertoli cell nuclei did not differ from week nine onwards and compared to CG.

Overall, apart from CX43 protein and mRNA, no investigated BTB components differed significantly between week twelve and CG. This almost complete recovery of the BTB in week twelve is in line with the first spermatozoa being observed between seven and twelve weeks after deslorelin implant removal [57], followed by normalized semen parameters up to week 19. Although weeks 15, 18, 21, and 24 were excluded from the analysis, a full stabilization of CX43 expression, which is essential for spermatogenesis [12], is expected in this interval.

### 4.2. Status of the Downregulated Testis Using Three Different GnRH-Agonist Implants and the Situation in Juvenile Testis

Besides well characterized histomorphological alterations (arrest of spermatogenesis, altered compartment composition) [37,38], as well as altered gene and protein expressions [38,39,40,58], downregulation significantly impacts Sertoli cell morphology and function. Not only are Sertoli cell nuclei flatter, polygonal, and positioned close to the basal membrane [37], they are significantly smaller, similarly to seasonal breeders out of season [59,60], indicating an altered Sertoli cell function [21,49]. In addition, altered *OCLN* (downregulated) and *CLDN5* (upregulated in relation to CG) gene expressions, as well as CLDN11 and CX43 protein expressions and distribution, further support the hypothesis of Sertoli cell “dysfunction”. Whether the status achieved at downregulation resembles seasonal breeders out of season still needs to be established with the current controversial results available: Whereas low CX43 mRNA and protein levels were reported in minks [61]—similar to our observations—significantly increased *CLDN3*, *CLDN11,* and *OCLN* expressions were described in Djungarian hamsters out of season [21,34].

Of considerable interest are our findings in juvenile testis: Interestingly, *CLDN5* was higher in JG compared to CG, supporting its relevance not only for the blood epithelial barrier but also for the BTB. Whether the relative enrichment of Sertoli cells might also play a role requires further investigation. On the contrary, *OCLN* gene expression was barely detectable and CLDN11 protein expression was absent in JG, indicating that both do not seem to be important at this developmental stage. The low *CX43* mRNA expression associated with a continuous weak staining in the Sertoli cell cytoplasm, mainly close to the basal membrane, stresses the functional importance of CX43 in JG. This observation is in good agreement with freeze-fracture studies in early postnatal canine testes where only few gap junctions were found [62,63]; similarly, Rüttinger et al. [3] found only weak CX43 staining in prepubertal testes of dogs 4–5 months old.

Comparing the downregulated and juvenile testes, this study further supports that downregulation is not equal to immaturity and that during recrudescence, puberty will not be repeated, as previously shown for various parameters [38,39,40,58]. As mentioned above, the significantly higher *CLDN5* expression during downregulation compared to JG and CG deserves further investigations, also on protein level. In general, our results rather indicate that spermatogenesis and the investigated BTB junctions are ready to be “re-activated”, as they are all present.

Nevertheless, the present data identified significant effects of groups, but no significant expression differences between treatments, confirming the consistent mode of action for the three GnRH agonists—azagly-nafarelin, buserelin acetate, and deslorelin—as described earlier [38,39,40,58]. Differences in GnRH agonist action were, however, observed in clinical studies depending on the dosage and the type of GnRH agonist, as well as between individual animals, in regard to, e.g., onset and duration of efficacy, as well as extent of downregulation [37,64,65]. As various GnRH agonists might differ in terms of suppression of LH and FSH, serum levels of both gonadotropins, as well as transcriptomic and proteomic investigations on isolated Sertoli cells, potentially accomplished by cell culture experiments, seem desirable for a better understanding of GnRH agonists, but also of Sertoli cell function [51,66].

## 5. Conclusions

This study provides valuable insights into the role of different BTB junctions in juvenile, downregulated, and adult canine testes and describes stage-specific CX43 protein expression. Long-term gonadotropin suppression by application of a slow-release GnRH-agonist implant not only induced testicular downregulation in the dog, but also disruption of the BTB, independent of the GnRH agonist used. Sertoli cell nuclear area was reduced, as was *OCLN* expression, while *CLDN3* and *5* gene expressions were increased. These alterations, however, did not mimic a prepubertal status, in which the gene expression was even more decreased. Following abolishment of treatment, the effects on the BTB were reversible, although with differences to studies investigating seasonal breeders. To what extent the altered BTB during downregulation possibly vulnerates spermatogenesis to reprotoxic drugs or infection deserves further and functional investigations.

## Figures and Tables

**Table 1:** Study design. Blood–testis barrier (BTB) formation was studied in Dataset 1: recrudescence of spermatogenesis following removal of a slow-release GnRH-agonist implant containing azagly-nafarelin [37], and Dataset 2: the state of downregulation by different implants and controls [38].

**Dataset 1** **Recrudescence**	**Group Name**	**Week After Implant Removal**	**Recovery from** **Azagly-Nafarelin** **Implant** **Spermatogenic Stage (Most Developed Germ Cells)**	***n***
	W0	0	primary spermatocytes, no tubular lumen	3
	W3	3	round and elongating spermatids, tubular lumen reestablished	3
	W6	6	elongating spermatids	4
	W9	9	elongating and elongated	3
	W12	12	full spermatogenesis	3
	CG	No implant	adult untreated control dogs	5
**Dataset 2** **downregulation**	**Group name**	**Week after implant removal**	**Comparison of different** **GnRH slow-release implants**	***n***
	W0	0	dogs treated with azagly-nafarelin (Gonazon^®^) = week 0	3
	PG	0	dogs treated with buserelin acetate (Profact^®^ Depot)	3
	SG	0	dogs treated with deslorelin (Suprelorin^®^)	3
	CG	No implant	adult untreated control dogs	5
	JG	No implant	juvenile untreated dogs	3

**Table 2:** Sequences of primers for RT-qPCR, amplicon length, efficiency, and accession number (for = forward, rev = reverse, bp = base pairs).

	Oligonucleotide Sequence (5′–3′)	Amplicon Length (bp)	Efficiency	Accession Number
*OCLN*	for: TCCTGCTCGTCCTGAAGATC rev: TGTCCCCACCGTACACATC	138	1.96	NM_001003195
*CLDN3*	for: CATCGGCAGCAGCATCA rev: GCACCACGCAGTTCATCC	65	1.87	NM_001003088
*CLDN5*	for: GTGACCGCCTTCCTG rev: GCACGACATCCACAGCC	69	2.01	NM_001130861.1
*CLDN11*	for: CCACGGGGCTGTACCACT rev: CGAGGCTGCGATCATCAG	71	1.86	XM_003434149
*CX43*	for: CCATCTCTAACTCCCATGCACAGC rev: TGGCACGACTGCTGGCTCTGCT	137	1.84	NM_001002951
*GAPDH*	for: GGCCAAGAGGGTCATCATCTC rev: GGGGCCGTCCACGGTCTTCT	228	1.83	NM_001003142.1

**Table 3:** Claudin 11 and CX43 protein expression during recrudescence (Dataset 1) and downregulation (dataset 2) of spermatogenesis. Week (w) 0–12 compared to adult controls (CG), or different GnRH slow-release implants (W0, PG, and SG) compared to juvenile dogs (JG) and (CG); evaluated in 20 testicular tubules: mean gray scale values ($\bar{x}$ (SD)) and percentage of immunopositive area (PIA; %; modified mean and range of mean ± standard deviation, data was arc sine-transformed to achieve normal distribution, means and SD calculated and data retransformed), different superscripts indicate significant differences between groups within column (Tukey–Kramer test, *p* < 0.05).

**Dataset 1:** **Recrudescence**	**Group**	**PIA** **CLDN11**	**Mean Gray Scale CLDN11**	**PIA** **CX43**	**Mean Gray Scale CX43**
	W0	0.13 (0.03–0.30)	59.60 ± 0.82	6.36 (3.28–10.38) ^a,b^	153.92 ± 0.14 ^a^
	W3	0.45 (0.08–1.12)	59.78 ± 1.22	14.03 (11.82–16.40) ^a^	153.71 ± 0.50 ^a^
	W6	2.54 (0.29–6.91)	60.93 ± 0.59	5.84 (3.83–8.23) ^a,b^	151.24 ± 0.55 ^b^
	W9	2.12 (1.06–3.53)	61.33 ± 0.17	4.55 (1.10–10.20) ^a,b^	152.05 ± 1.52 ^a,b^
	W12	1.81 (0.88–3.07)	60.72 ± 0.36	1.43 (0.38–3.14) ^b^	151.59 ± 1.41 ^b^
	CG	3.42 (0.83–7.69)	61.17 ± 1.18	2.57 (1.16–4.51) ^b^	153.30 ± 0.54 ^a^
	*p*-value (ANOVA)	n.s.	n.s.	0.0021	0.0028
**Dataset 2:** **downregulation**	**Group**	**PIA** **CLDN11**	**Mean gray scale CLDN11**	**PIA** **CX43**	**Mean gray scale CX43**
	W0	0.13 (0.03–0.30) ^a,b^	59.60 ± 0.82 ^a^	6.36 (3.28–10.38) ^a,b^	153.92 ± 0.14
	PG	2.20 (0.37–5.51) ^a,b^	61.65 ± 2.05 ^a^	1.39 (0.84–2.06) ^a^	153.43 ± 0.50
	SG	0.72 (0.14–1.76) ^a,b^	59.34 ± 1.47 ^a^	2.86(0.90–5.88) ^a,b^	153.36 ± 0.54
	JG	0.00 (0.00–0.00) ^a^	56.00 ± 0.00 ^b^	9.13 (5.06–14.24) ^b^	153.50 ± 0.22
	CG	3.42 (0.83–7.69) ^b^	61.17 ± 1.18 ^a^	2.57 (1.16–4.51) ^a^	153.30 ± 0.54
	*p*-value (ANOVA)	0.0179	0.0008	0.016	n.s.

n.s. not significant.

## Data Availability

The original contributions presented in this study are included in the article/Supplementary Materials. Further inquiries can be directed to the corresponding author.

## Supplementary Materials

The following supporting information can be downloaded at: https://www.mdpi.com/article/10.3390/ani16020254/s1, Figure S1: Immunostaining for Claudin 11 in dog testicular tissue dataset 1. A: group W0, B: group W3, C: group W6, D: group W9, E: group W12, F: CG (control group) G (insert): negative control (all magnification: × 400, scale bar = 20 µm). ⌂ Sertoli cell, ▲ spermatogonia, ∆ primary spermatocyte, ↑ elongating spermatids; Figure S2: Immunostaining for Claudin 11 in dog testicular tissue dataset 2. A: group W0, B: PG; C: SG, D: JG (juvenile), E: CG (control group), F: negative control (all magnification: × 400, scale bar = 20 µm). ⌂ Sertoli cell, ▲ spermatogonia, ∆ primary spermatocyte, ↑ elongating spermatids; Figure S3: Immunostaining for Connexin 43 in dog testicular tissue dataset 1. A: group W0, B: group W3, C: group W6, D: group W9, E: group W12, F: CG (control group), G (insert): negative control (all magnification: × 400, scale bar = 20 µm). ⌂ Sertoli cell, ▲ spermatogonia, ∆ primary spermatocyte, ↑ elongating/elongated spermatids; Figure S4: Immunostaining for Connexin 43 in dog testicular tissue dataset 2. A: group W0, B: PG, C: SG, D: JG (juvenile), E: CG (control group), F: negative control (all magnification: × 400, scale bar = 20 µm). ⌂ Sertoli cell, ▲ spermatogonia, ∆ primary spermatocyte, ↑ elongating spermatids.

## Abbreviations

The following abbreviations are used in this manuscript:

BTB	Blood–testis barrier
CX43	Gap junctional protein connexin 43
OCLN	Occludin
CLDN	Claudin
JAM	Junctional adhesion molecule
FSH	Follicle stimulating hormone
GnRH	Gonadotropin-releasing hormone
CG	Control group
W	Week of castration/Gonazon^®^ group
PG	Profact^®^ group
SG	Suprelorin^®^ group
JG	Juvenile group
GAPDH	Glyceraldehyde-3-phosphate dehydrogenase
PIA	Percentage immunopositive area
SD	Standard deviation
ANOVA	Analysis of variance
