# Supplementary material for: Testicular Gap (CX43) and Tight Junction (OCLN, CLDN3, 5 and 11) Components in the Dog Are Affected by GnRH-Mediated Downregulation
Source: Animals (Basel). 2026 Jan 14;16(2):254. doi: 10.3390/ani16020254 (PMC12837560; doi:10.3390/ani16020254)
Supplement: Supplementary file 1 [file animals-16-00254-s001.zip › animals-4031011-supplementary.pdf]

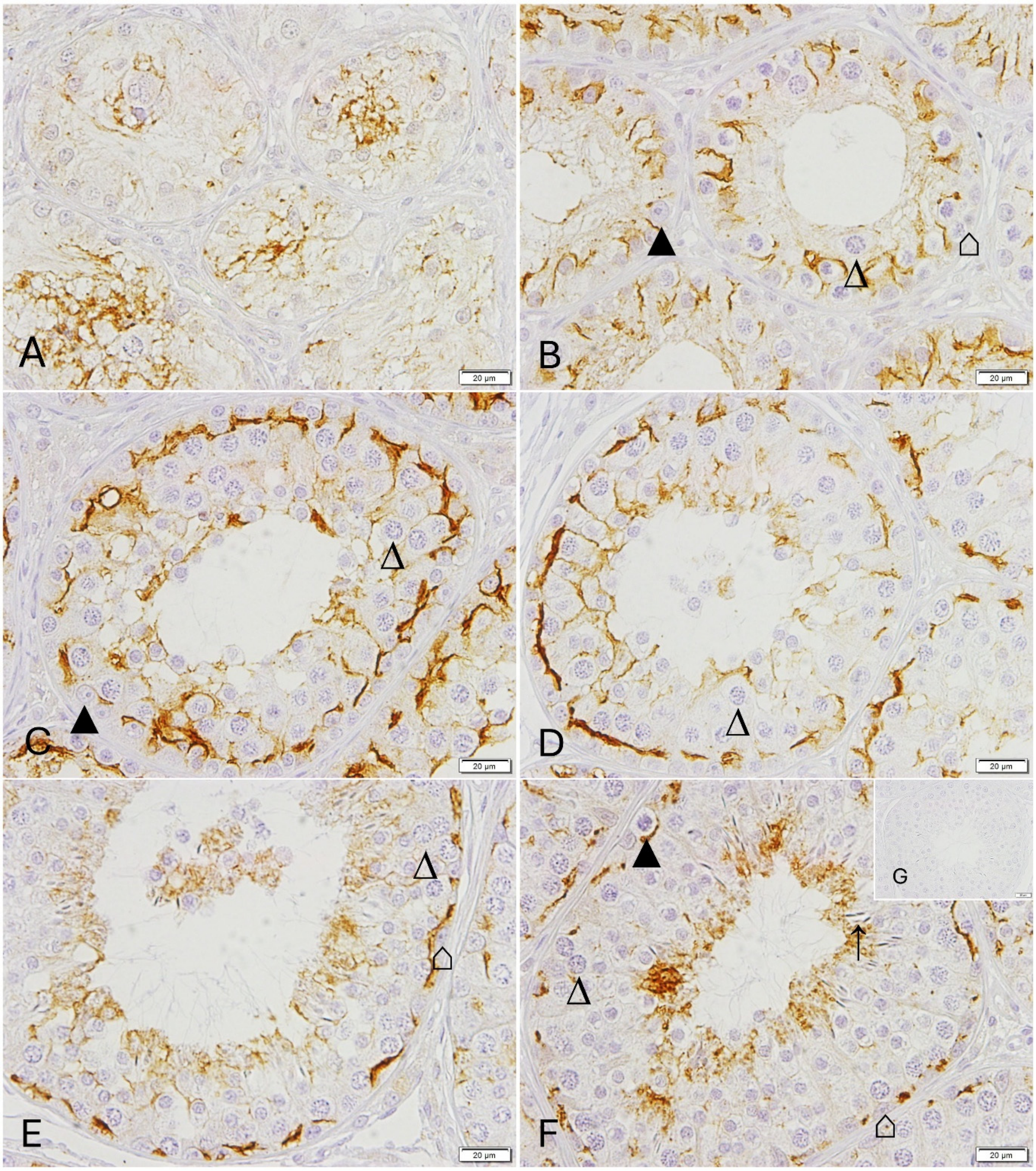

Figure S1: Immunostaining for Claudin 11 in dog testicular tissue dataset 1. A: group W0, B: group W3, C: group W6, D: group W9, E: group W12, F: CG (control group) G (insert): negative control (all magnification: x 400, scale bar = 20 µm). ⌂ Sertoli cell, ▲ spermatogonia, ∆ primary spermatocyte, ↑ elongating spermatids

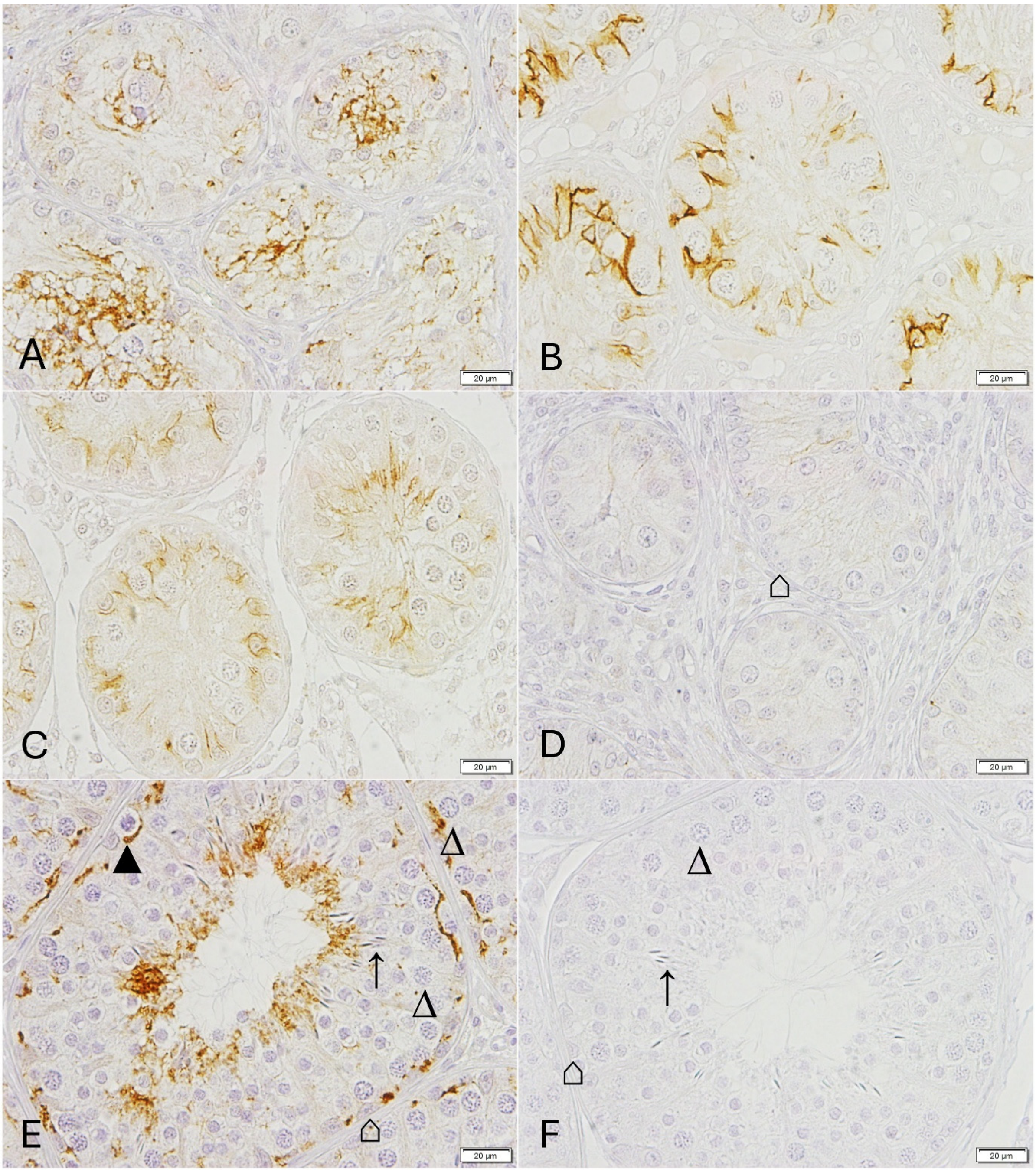

Figure S2: Immunostaining for Claudin 11 in dog testicular tissue dataset 2. A: group W0, B: PG; C: SG, D: JG (juvenile), E: CG (control group), F: negative control (all magnification: x 400, scale bar = 20 µm). ⌂ Sertoli cell, ▲ spermatogonia, ∆ primary spermatocyte, ↑ elongating spermatids

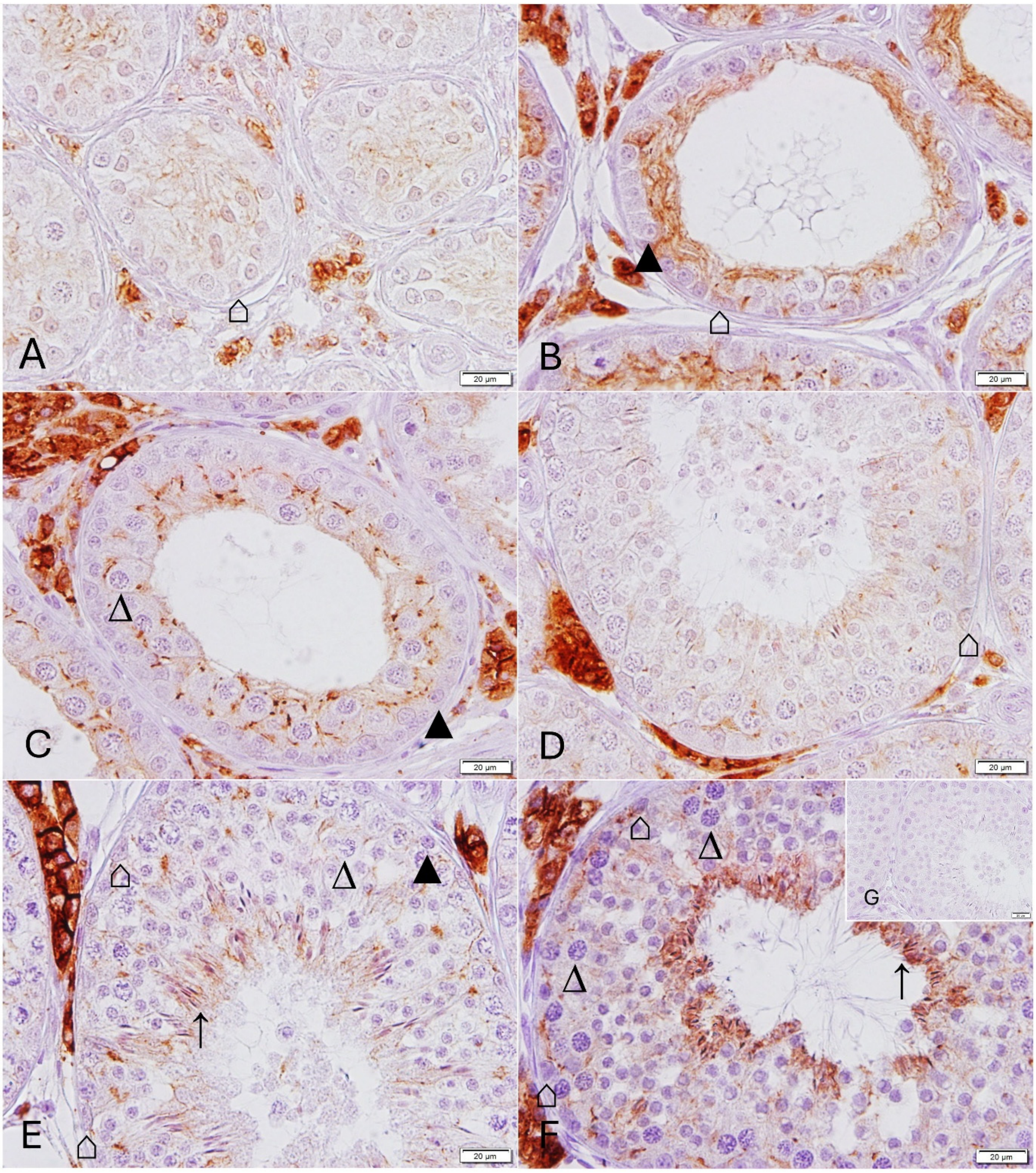

Figure S3: Immunostaining for Connexin 43 in dog testicular tissue dataset 1. A: group W0, B: group W3, C: group W6, D: group W9, E: group W12, F: CG (control group), G (insert): negative control (all magnification: x 400, scale bar = 20 µm). ⌂ Sertoli cell, ▲ spermatogonia, ∆ primary spermatocyte, ↑ elongating/elongated spermatids

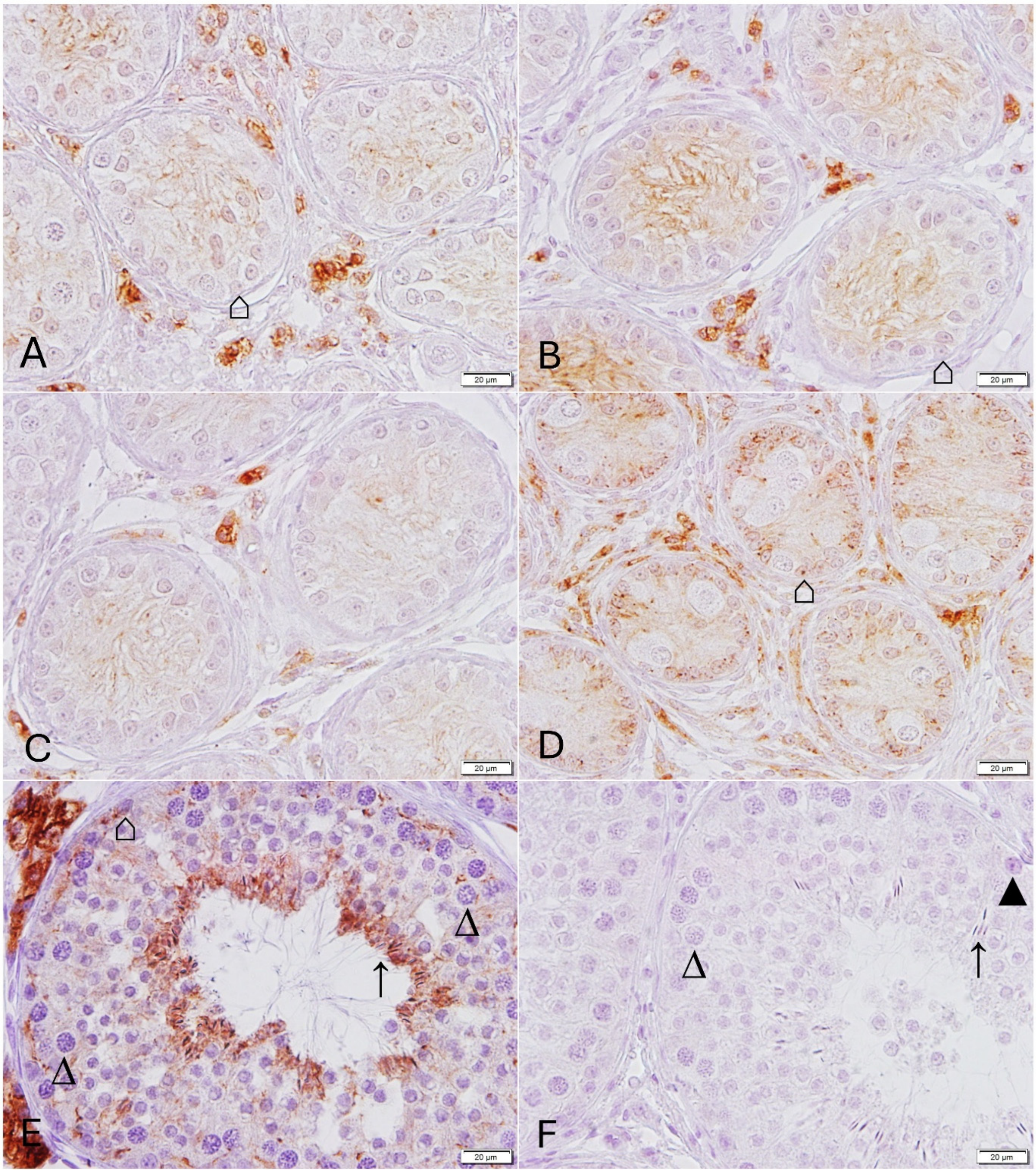

Figure S4: Immunostaining for Connexin 43 in dog testicular tissue dataset 2. A: group W0, B: PG, C: SG, D: JG (juvenile), E: CG (control group), F: negative control (all magnification: x 400, scale bar = 20 µm). ⌂ Sertoli cell, ▲ spermatogonia, ∆ primary spermatocyte, ↑ elongating spermatids
